# Boosting maternal and neonatal anti–SARS-CoV-2 humoral immunity using a third mRNA vaccine dose

**DOI:** 10.1172/jci.insight.158646

**Published:** 2023-01-10

**Authors:** Adva Cahen-Peretz, Lilah Tsaitlin-Mor, Hadas Allouche Kam, Racheli Frenkel, Maor Kabessa, Sarah M. Cohen, Michal Lipschuetz, Esther Oiknine-Djian, Sapir Lianski, Debra Goldman-Wohl, Asnat Walfisch, Michal Kovo, Michal Neeman, Dana G. Wolf, Simcha Yagel, Ofer Beharier

**Affiliations:** 1 Department of Obstetrics and Gynecology, Hadassah Hebrew University Medical Center, Jerusalem, Israel.; 2 Clinical Virology Unit, Hadassah Medical Center, Hebrew University of Jerusalem, Jerusalem, Israel.; 3 Lautenberg Center for General and Tumor Immunology, Faculty of Medicine, Hebrew University of Jerusalem, Jerusalem, Israel.; 4 Department of Obstetrics and Gynecology, Meir Medical Center, Kfar Saba, Israel.; 5 Department of Biological Regulation, Weizmann Institute of Science, Rehovot, Israel.

**Keywords:** COVID-19, Vaccines, Adaptive immunity, Epidemiology, Obstetrics/gynecology

## Abstract

**BACKGROUND.:**

To minimize COVID-19 pandemic burden and spread, 3-dose vaccination campaigns commenced worldwide. Since patients who are pregnant are at increased risk for severe disease, they were recently included in that policy, despite the lack of available evidence regarding the impact of a third boosting dose during pregnancy, underscoring the urgent need for relevant data. We aimed to characterize the effect of the third boosting dose of mRNA Pfizer BNT162b2 vaccine in pregnancy.

**METHODS.:**

We performed a prospective cohort study of anti–SARS-CoV-2 antibody titers (*n* = 213) upon delivery in maternal and cord blood of naive fully vaccinated parturients who received a third dose (*n* = 86) as compared with 2-dose recipients (*n* = 127).

**RESULTS.:**

We found a robust surge in maternal and cord blood levels of anti–SARS-CoV-2 titers at the time of delivery, when comparing pregnancies in which the mother received a third boosting dose with 2-dose recipients. The effect of the third boosting dose remained significant when controlling for the trimester of last exposure, suggesting additive immunity extends beyond that obtained after the second dose. Milder side effects were reported following the third dose, as compared with the second vaccine dose, among the fully vaccinated group.

**CONCLUSION.:**

The third boosting dose of mRNA Pfizer BNT162b2 vaccine augmented maternal and neonatal immunity with mild side effects. These data provide evidence to bolster clinical and public health guidance, reassure patients, and increase vaccine uptake among patients who are pregnant.

**FUNDING.:**

Israel Science Foundation KillCorona grant 3777/19; Research grant from the “Ofek” Program of the Hadassah Medical Center.

## Graphical abstract

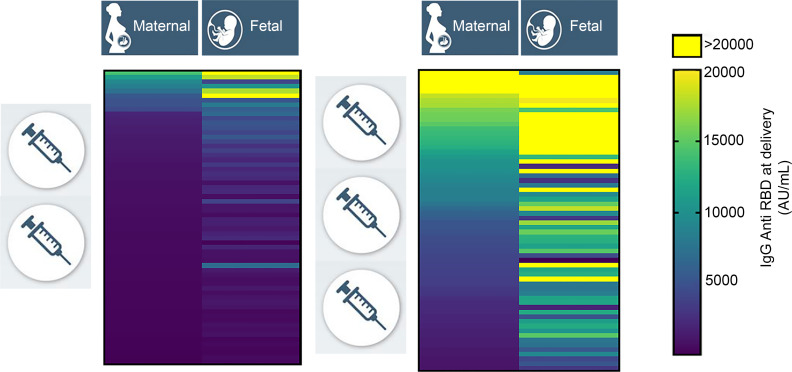

## Introduction

As the COVID-19 pandemic continues to evolve (1), waning in anti–SARS-CoV-2 antibody titers (2) and the appearance of novel variants challenge immunity acquired following primary infection or vaccination (3). Data from patients who are not pregnant show that the boosting of waning immunity via a single boosting dose of an mRNA vaccine greatly enhances protection against reinfection and novel variants (4), including the B.1.617.2 (Delta) (5) and the B.1.1.529 (Omicron) (6) variants.

Increased risk for severe illness, mechanical ventilation, and death from COVID-19 has been reported in individuals who are pregnant compared with properly matched nonpregnant cohorts (7–19). Maternal COVID-19 morbidity and pregnancy-related complications also significantly affect fetal and neonatal health (20–22).

Maternal anti–SARS-CoV-2 antibodies are an important module of maternal antiviral immunity. Additionally, vertical transport of maternal IgG across the human placenta provides maternally derived antibody to the neonate, hence providing the first line of defense for neonatal humoral immunity. Importantly, recent reports show the protective effect of anti–COVID-19 vaccination during pregnancy in reducing maternal infection and severe illness (23–25).

In an attempt to stop the COVID-19 pandemic burden, 3-dose vaccination campaigns commenced worldwide. Israel was the first country (July 2021) to launch an unprecedented third dose booster vaccination campaign, using a single dose of Pfizer BNT162b2 mRNA vaccines. Since patients who are pregnant are at increased risk for severe disease, they were urged to attend for inoculation, despite the lack of available evidence regarding the impact of a third boosting dose during pregnancy (26). Similar recommendations were recently adopted by the American Centers for Disease Control and Prevention (CDC) and the American College of Obstetrics and Gynecologists (ACOG) (27, 28). These recommendations were based on the best available evidence, extrapolating data from nonpregnant populations. Nevertheless, pregnancy is a time of immune system modulation, affecting many aspects of the maternal immune response, including the humoral response (29, 30). Lack of relevant data creates conditions amenable to the dissemination of disinformation and vaccine hesitancy among patients who are pregnant. Clinicians around the world face daily questions and concerns regarding boosting during pregnancy. Consequently, compliance among gravidae in numerous countries and societies is low (31–33), despite their increased risk, amidst persistent patient concerns regarding the necessity and potential impact of vaccination and boosting during pregnancy (33–36). This situation underscores the urgent need for relevant evidence regarding vaccine boosting during pregnancy.

Evidence-based answers to questions often posed by health care providers, patients, and families are lacking. Currently, it is unclear whether a third boosting dose during pregnancy will boost maternal and newborn immunity, whether boosting induces immunity over and above that of the standard 2-dose regimen, and whether the timing of initial vaccination (before or during pregnancy) will affect the response to boosting. Concerns have been expressed regarding the side effects of the third boosting dose and whether increased number or intensity of side effects points to a more robust immune response.

Here, we aimed to provide relevant data regarding the impact of a third, boosting dose of Pfizer BNT162b2 mRNA vaccine on maternal and neonatal antibody titers, and we characterize the side effect profile of a third anti–COVID-19 booster during pregnancy.

## Results

### Participant characteristics.

**Figure 1:**
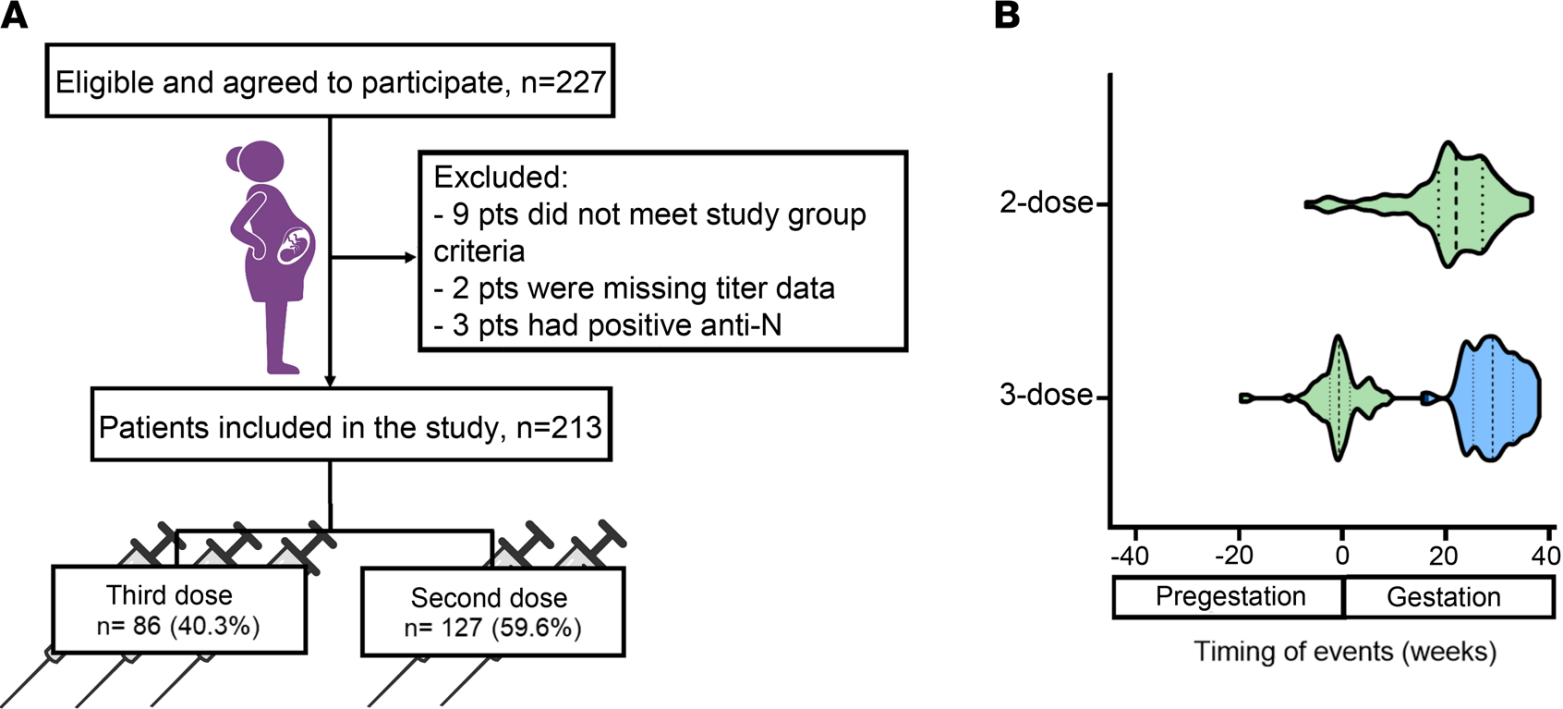
Flow chart and Patient characteristics. (**A**) Schematic flow chart of patients included in the study. (**B**) Violin plot showing the timing of vaccination received before delivery in 2-dose recipients (top, *n* = 86) and third-dose patients (bottom, *n* = 127). Blue violin shows the timing of the third dose; green violins show the timing of the second dose in both study groups. Negative x axis values indicate vaccination received prior to pregnancy. Dashed lines indicate median. Dotted lines indicate interquartile range.

The study consisted of 213 parturients presenting for delivery at Hadassah Medical Center, Mt. Scopus campus, Jerusalem, Israel, as detailed in Figure 1A. It is important to note that 3 patients were positive for anti-N (anti-nucleocapsid, evidence of recent or prior COVID-19 infection); therefore, they were excluded from the study. Another 8 samples could not be analyzed for anti-N due to the small amount of serum stored. The demographic and medical characteristics of the study groups, as well as median time from last exposure (i.e., third boosting vaccine dose or second vaccine dose) to delivery, are shown in Table 1. Obstetric clinical parameters and neonatal outcomes did not differ among the groups (including fetal sex) except for the rate of diabetes. Higher rates of diabetes (pregestational and gestational) were found in the third dose group as compared with 2 dose recipients (*P* = 0.003). Importantly, 6 of 8 patients received the third dose following diabetes diagnosis, as compared with 1 patient in the 2-dose recipients group who was also diagnosed before the second vaccine dose. Moreover, mean maternal age of patients diagnosed with diabetes was slightly higher, as compared with the total group (32.9 versus 29.9, *P* = 0.130). Violin plots illustrate the distribution of exposure timing prior to and through the course of gestation, in both study groups (Figure 1B).

### Maternal serological response to a third boosting dose of mRNA BNT162b2 vaccine.

**Figure 2:**
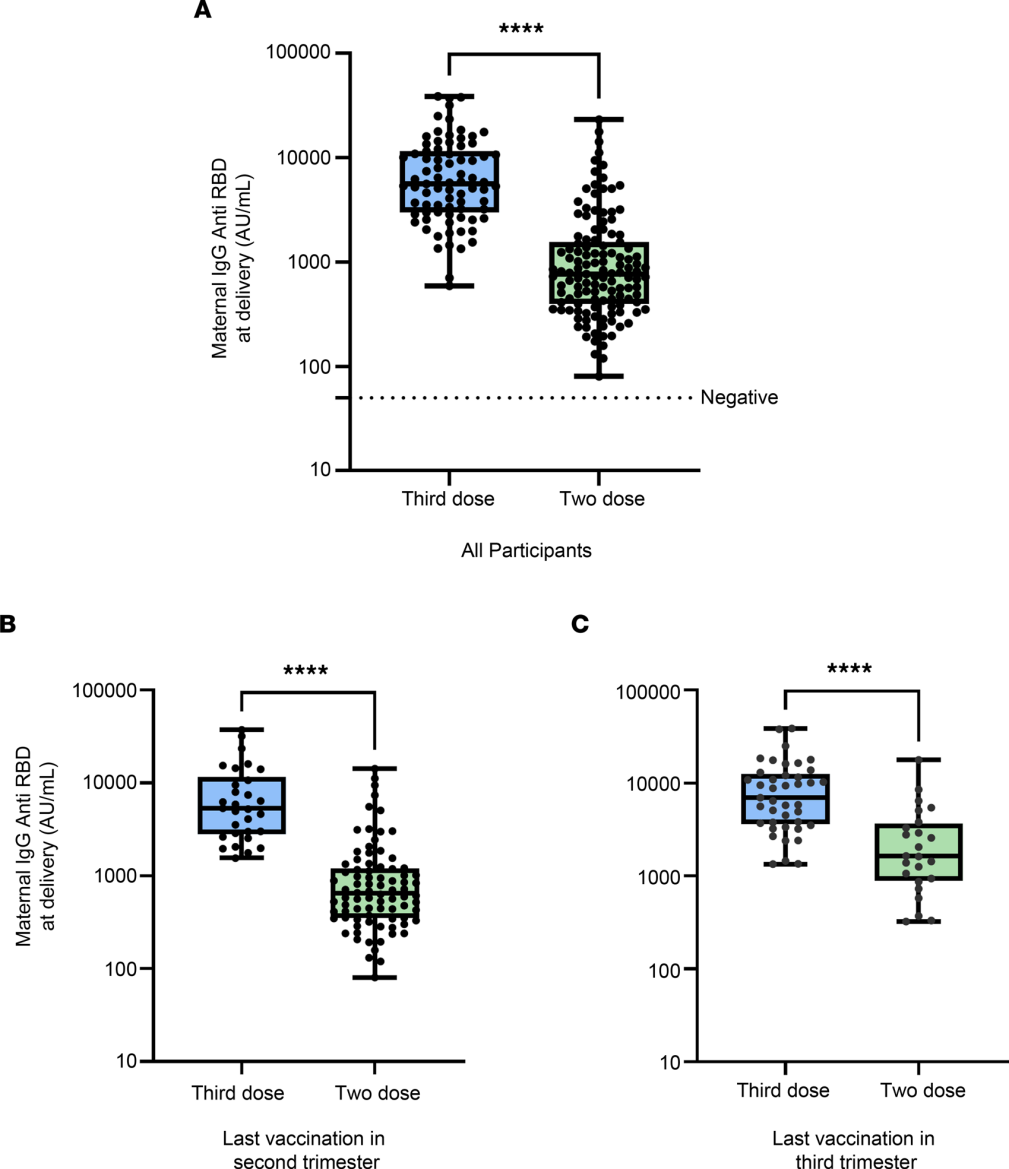
Third boosting dose of BNT162b2 mRNA vaccine administered during pregnancy produced a vigorous surge in anti–SARS-CoV-2 antibody titers detected at delivery. Blue indicates participants that received a third, booster dose during pregnancy (*n* = 79; sera samples were missing for 7 women in the 3-dose group) . Green indicates 2-dose recipients (*n* = 127). (**A**) Comparison of maternal anti–SARS-CoV-2 antibody titers. The horizontal dotted line indicates a titer below 50 (negative result). (**B** and **C**) Comparison of maternal anti–SARS-CoV-2 antibody titers between third boosting dose group and 2-dose recipients group, for parturients who received their last vaccination dose in the second (**B**) or third (**C**) trimesters of pregnancy (3-dose, *n* = 30; 2-dose, *n* = 84, in **B**; 3-dose, *n* = 41, 2-dose, *n* = 24, in **C**). Between-group comparisons were analyzed using the Mann Whitney *U* test; ****P < 0.0001.

Figure 2A presents anti–SARS-CoV-2 antibody levels in maternal blood, for the study groups. We observed significantly higher antibody titers upon delivery in patients who received a third boosting dose of mRNA vaccine as compared with the 2-dose recipients (7.3-fold) (Figure 2A and Supplemental Figure 1; supplemental material available online with this article; https://doi.org/10.1172/jci.insight.158646DS1). In order to control for the interval from vaccination to delivery, and to determine whether higher titers reflected a stronger humoral immune response or mirrored a shorter interval from exposure to delivery, groups were stratified by trimester of vaccination. Our data show that anti–SARS-CoV-2 antibody titers were significantly higher following the third booster, when compared with the second dose of vaccine, given at the same trimesters (Figure 2, B and C). When maternal titer was stratified by maternal age in each group, we found no correlation (*r* = 0.143 and –0.003, *P* = 0.207 and 0.967, for third and second dose group, respectively). Our data reveal an additional significantly robust increase in maternal humoral response to the third boosting dose.

We next aimed to determine whether the timing of initial vaccination affects maternal humoral response to the boosting dose. We found that vaccination that occurred before versus during pregnancy (Supplemental Figure 2) did not alter maternal humoral response, as detected by anti–SARS-CoV-2 titers at the time of delivery. Additionally, we assessed the absolute time interval between 2 and 3 doses (irrespective of pregnancy) and its association with maternal titers. We found no correlation between the time interval of the second and third dose and the maternal antibody titer (*r* = –0.090, *P* = 0.430; Supplemental Figure 3).

### Transplacental antibody transfer following a third vaccine dose.

**Figure 3:**
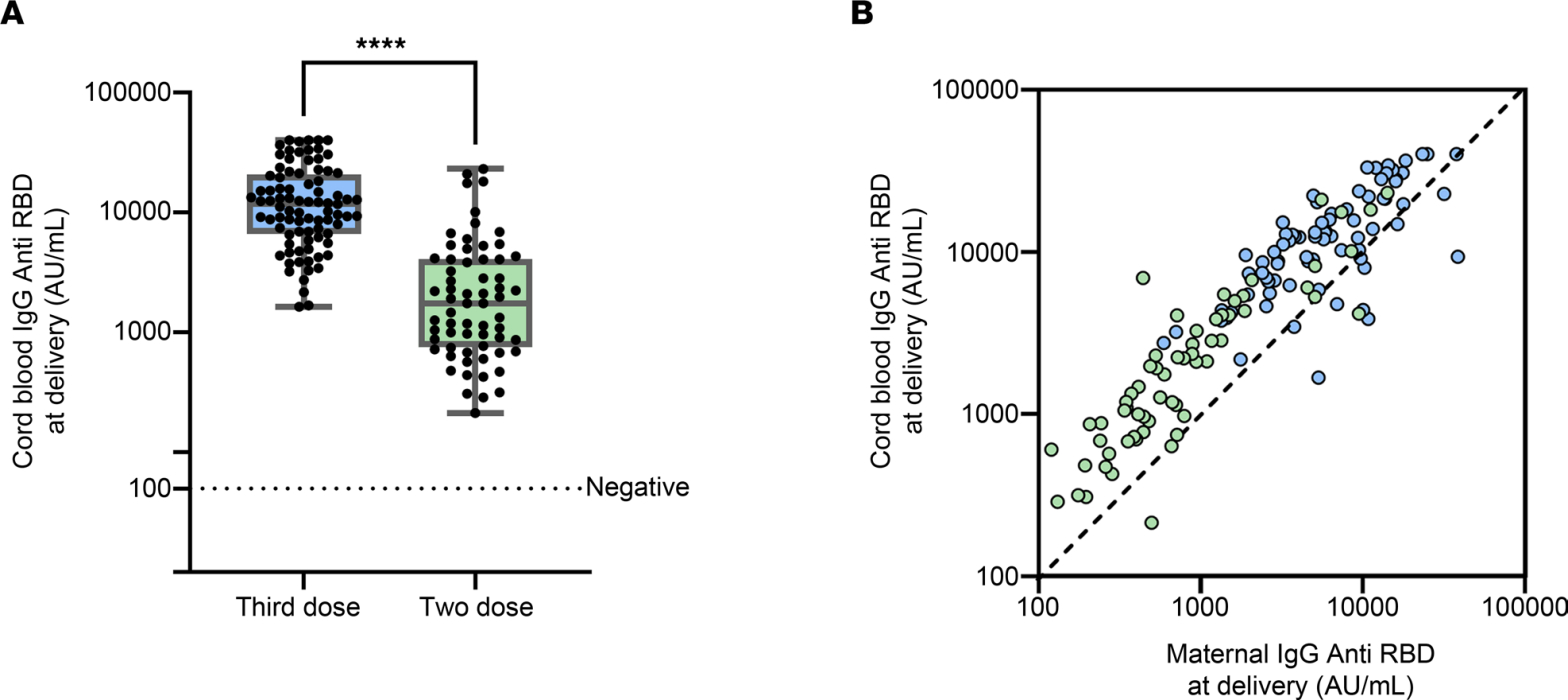
Third boosting dose administered during pregnancy produced a vigorous surge in anti–SARS-CoV-2 antibody titers detected in cord blood. Blue indicates participants who received a third, boosting vaccine dose during pregnancy (*n* = 85; sera samples were missing for 1 newborn from the 3 dose group and 63 from the 2-dose group). Green indicate 2-dose recipients (*n* = 64; sera samples were missing for 1 newborn from the 3 dose group and 63 from the 2-dose group). (**A**) Between-group comparison of cord blood anti–SARS-CoV-2 antibody titers. The horizontal dotted line indicates a titer below 50 (negative result). Differences between the groups were analyzed using the Mann Whitney *U* test; ****P < 0.0001. (**B**) Correlation between anti–SARS-CoV-2 antibody titers in maternal/cord blood dyads (3-dose, *n* = 78, blue circles; 2-dose, *n* = 64, green circles) was analyzed with Spearman’s correlation test. The diagonal line represents a geometric 1:1 ratio. Anti–SARS-CoV-2 antibody titers in cord blood positively correlated with maternal anti–SARS-CoV-2 concentrations (3-dose, *r* = 0.745, *P* < 0.0001; 2-dose, *r* = 0.878, *P* < 0.0001).

Figure 3A presents anti–SARS-CoV-2 antibody levels in the cord blood of the study groups. Cord blood titers were significantly higher following the third boosting dose compared with the full-vaccination regimen (Figure 3A). Maternal and cord blood anti–SARS-CoV-2 titers were measured in 78 maternal/cord blood dyads of participants who received the third boosting dose, to assess transplacental vertical transmission. Anti–SARS-CoV-2 antibody titers were found to be positively correlated (*r* = 0.745 and 0.878, *P* < 0.0001 and *P* < 0.001, for third and second dose recipients, respectively) and significantly higher (*P* < 0.0001 for both groups) in cord blood compared with maternal blood, as shown in Figure 3B. These findings support the notion that maternal protective immune response to the third dose is efficiently transmitted to the fetus, leading to significantly higher protective antibody titers for the newborn. Moreover, we assessed cord/maternal titer ratio stratified by the time interval between vaccination and delivery. Among third-dose patients, we found significant positive correlation between cord/maternal titer ratio and the time interval between vaccination and delivery (*r* = 0.359, *P* = 0.001). We found no correlation among second-dose recipients (*r* = –0.149, *P* = 0.238; Supplemental Figure 4).

### Reported vaccination side effects and maternal anti–SARS-CoV-2 antibody titers.

**Figure 4:**
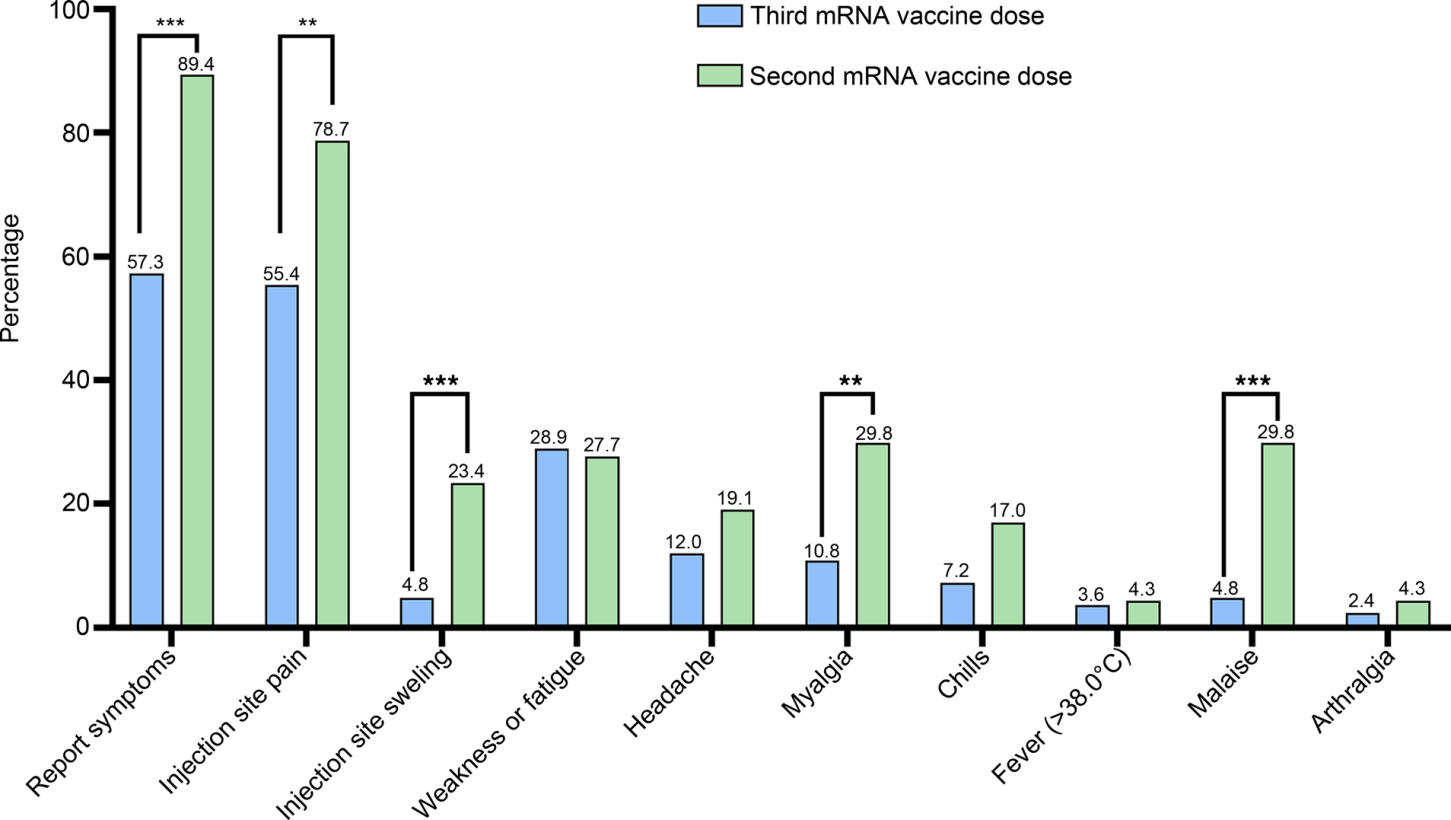
Comparison of the most frequent side effects reported by participants following the third, boosting vaccine dose (blue) versus 2-dose recipients (green). Data are presented as proportion of reported, frequent side effects among participants. Data were collected before or following labor using a detailed standard questionnaire. Differences between groups were analyzed by the Pearson χ^2^ analysis, with Bonferroni correction for multiple comparisons. Seventy-six patients from the 3-dose group answered the questionnaire, and 47 patients from the 2-dose group answered the questionnaire. **P < 0.01, ***P < 0.001.

**Figure 5:**
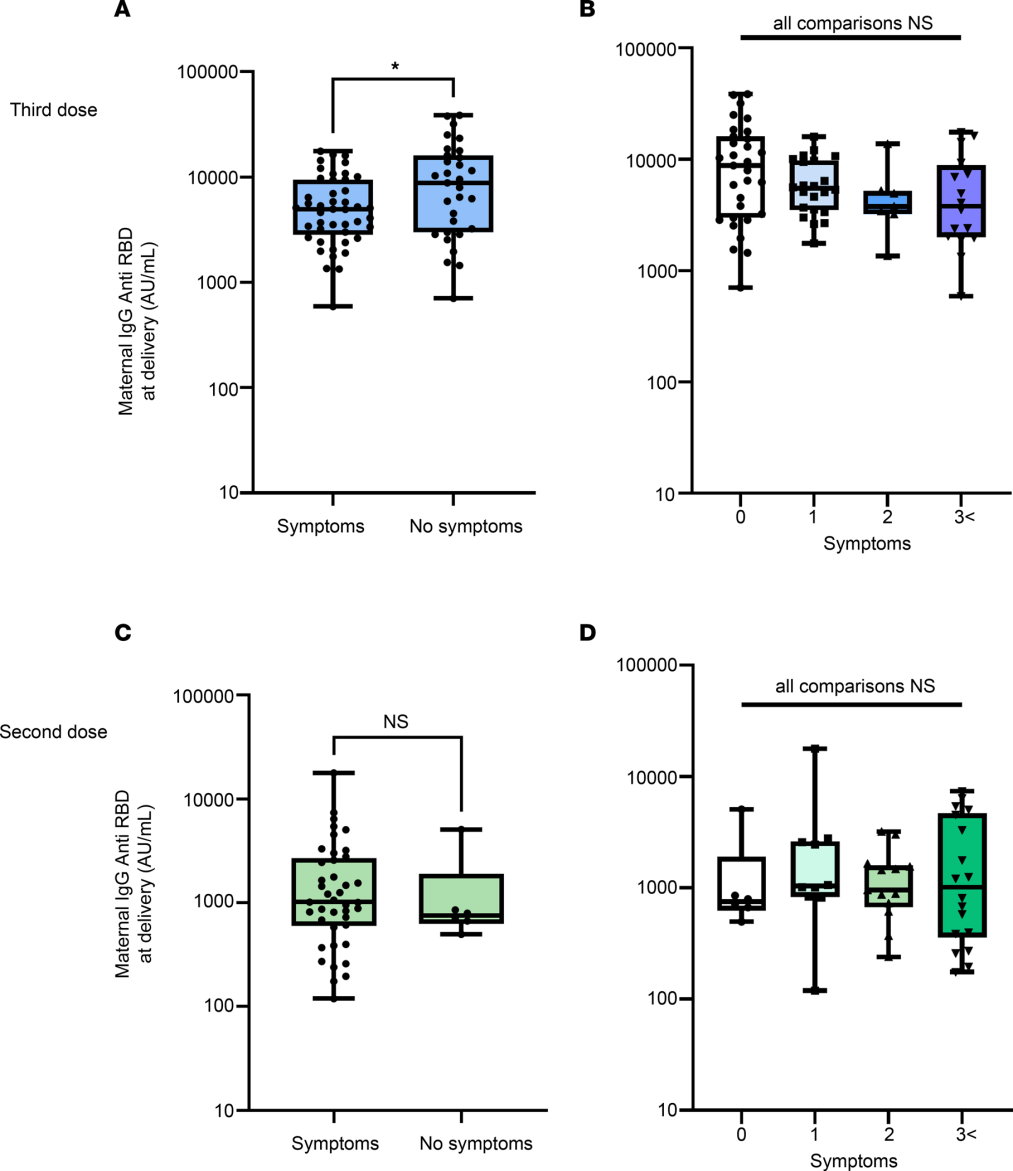
Maternal anti–SARS-CoV-2 antibody titers at delivery and symptoms following vaccination. Blue indicates participants who received a third booster dose. Green indicates 2-dose recipients. (**A** and **B**) Comparison of maternal anti–SARS-CoV-2 antibody titers for third booster, analyzed by presence (*n* = 45) or absence (*n* = 31) of reported symptoms (**A**) and analyzed by number of reported symptoms (**B**). (**C** and **D**) Comparison of maternal anti–SARS-CoV-2 antibody titers for second vaccination, analyzed by presence (*n* = 41) or absence (*n* = 6) of reported symptoms (**C**) and analyzed by number of reported symptoms (**D**). Significant differences for comparison were determined by Mann-Whitney *U* test and Kruskal-Wallis 1-way ANOVA, followed by Dunn’s correction for multiple comparisons. *P < 0.05.

We further characterized the side-effect profile of the third boosting dose. Among the third-dose group, 56.8% of patients reported 1 or more adverse related symptoms. This figure is significantly lower, as compared with the second-dose recipients (89.1%, *P* < 0.001). When comparing the duration of symptoms among groups, median time of symptoms was significantly lower among the third dose, as compared with the 2 dose recipients (1.0 versus 2.0 days, *P* < 0.001). Figure 4 illustrates the side effects reported following the 2 vaccination regimens. Overall, the third booster dose caused fewer side effects compared with the second dose, with lower rates of injection site pain and swelling, myalgia, and general malaise. Importantly, fewer than 5% of patients who received the third dose reported fever. Among our study groups, the presence and number of symptoms following vaccination were not correlated with increased maternal anti–SARS-CoV-2 titers at delivery (Figure 5). Unexpectedly, participants who reported no symptoms following the third booster had significantly higher maternal anti–SARS-CoV-2 titers (1.75-fold, *P* = 0.041), as compared with participants who reported side effects.

## Discussion

In the present study, we aimed to characterize the impact of the third mRNA Pfizer BNT162b2 boosting dose during pregnancy on maternal and cord blood antibody levels upon delivery. We found a robust surge in maternal and cord blood levels of anti SARS-CoV-2 titers at the time of delivery, when considering pregnancies in which the mother received a third boosting dose compared with 2-dose recipients. The observed effect of the third boosting dose remained significant when controlling for the trimester of last exposure, suggesting that additive immunity extends beyond that obtained after the second dose. Within our cohort, the third boosting dose resulted in a mild side-effect profile, with lower reported side effects as compared with the second dose of standard COVID-19 vaccination during pregnancy. The higher rates of diabetes among the third-dose group may be related to higher compliance to receive the additional booster, as most patients received the third dose after the diagnosis of diabetes. Another explanation might stem from the higher mean maternal age among patients diagnosed with diabetes. The CDC, ACOG, and others have recommended third boosting vaccine doses for fully vaccinated gravidae, to minimize the risks of severe disease (27, 28). However, vaccination uptake has been slow among gravidae in many societies (31–33) and may be even lower for booster shots, as concerns surrounding vaccination during pregnancy persist. In Israel, where boosting has been official policy for several months, health care providers and public health regulators face recurrent questions regarding the impact and need for vaccine boosting. In this context, our first reassuring data may serve clinicians and policymakers searching for relevant evidence to inform patients and increase vaccine boosting uptake.

In their recent paper, Yang et al. (37) demonstrated that anti–SARS-CoV-2 antibody levels fall rapidly in gravidae following vaccination, similar to the general population, and this fall may expose gravidae to reduced protection against severe illness. We demonstrate that a third, boosting dose of the BNT162b2 mRNA vaccine markedly increased anti–SARS-CoV-2 antibody titers in maternal and cord blood, likely conferring improved protection from infection in this vulnerable population. In addition, we found that the cord/maternal transfer ratio correlated between time interval of vaccination and delivery among the third-dose but not the second-dose recipients. These results could be explained by differences in duration from last vaccination to delivery between the 2 groups, suggesting that, in the early days following vaccination, the ratio was mainly affected by a rapid surge in maternal titers and inadequate catchup in cord titers. Later, the active transport via the placenta led to appropriate increase in cord titers, therefore increasing the transplacental ratio rate. Because the newborn depends on this humoral immunity as a primary line of defense against viral disease, these enhanced antibody titers likely provide more effective and longer-lasting protection against neonatal COVID-19 disease.

As the pandemic persists, more women will commence their pregnancy fully vaccinated. We show that providing a third dose in these patients results in a robust response, which is not affected by the timing of the initial vaccination, before or during pregnancy.

Caregiver recommendations regarding the third boosting dose are frequently met with questions regarding the common side-effect profile of this therapy during pregnancy. Our data are the first to our knowledge to show that, in general, the reported side effects of the third boosting dose are milder than those reported following the second dose. Specifically, there were lower rates of injection-site pain and swelling, myalgia, and general malaise. We can speculate that the difference in the presence and number of reported side effects may stem from recall bias or self-selection on the part of patients attending for the third boosting dose. Importantly, fewer than 5% of patients who received the third dose reported fever, which may be of concern as regards neonatal outcomes (38). Among our study groups, the presence and number of symptoms following vaccination were not associated with increased maternal anti–SARS-CoV-2 titers at delivery. These findings can provide reassurance to gravidae and their caregivers.

### Strengths and limitations.

The present study has several strengths. Its prospective design, recognized serological assays, blinded serum analysis, and standardized side-effect questionnaire, which is comparable with other studies, strengthen our findings. Although samples were collected from 1 center, a diverse population was recruited. Limitations include the relatively small patient numbers and lack of long-term follow-up of the newborns as regards the persistence of anti–SARS-CoV-2 antibody levels. We did not collect data regarding the magnitude and duration of fever. Finally, anti–SARS-CoV-2 antibody levels have been shown to correlate with protection against symptomatic breakthrough reinfection (39–41); however, immunological memory encompasses memory B cells, memory CD4^+^ T cells, and/or memory CD8^+^ T cells that may support protection (42, 43) but were not evaluated in the present study. Further studies are necessary to evaluate these components of the immunological response.

### Conclusions.

Administration of a third, boosting dose of Pfizer BNT162b2 mRNA vaccine provides a robust surge in anti–SARS-CoV-2 antibody titers in maternal and cord blood, with a mild side effect profile. These data are urgently needed by clinicians and policymakers that encounter questions and concerns regarding the benefits of a third boosting anti–SARS-CoV-2 vaccine dose during pregnancy, in order to provide evidence-based guidance.

## Methods

### Study population.

This was a prospective cohort study of parturients admitted for delivery from April 2021 to January 2022 at Hadassah Mt. Scopus Medical Center. Parturients were approached and invited to participate in a biorepository study following their admission to the delivery room. Eligibility criteria included willingness to participate and provide informed consent, age of 18–45 years, naive to COVID-19 infection, and anti–SARS-CoV-2 vaccination before or during pregnancy. Pregnant women with positive COVID-19 reverse transcription PCR at delivery were excluded from the study. Additionally, we performed an analysis for anti-N. Three patients were excluded due to positive anti-N results.

Following informed consent, participants were assigned to 1 of 2 study groups: (a) naive fully vaccinated parturients who received a third boosting dose of BNT162b2 mRNA (“third dose group”) or (b) 2-dose recipients parturients.

Participants had maternal and cord blood samples drawn at the time of delivery. Demographic, obstetric, and vaccine side-effect profile data were collected for all patients from the electronic medical record and from dedicated side-effect questionnaires. Each patient reported their last vaccination side effect or side effects (from either the second or third inoculation) retrospectively at the time of enrollment.

### Sample and data collection and handling.

Maternal and umbilical cord blood samples were collected from enrolled participants immediately following delivery. Blood samples were centrifuged at 1,000*g* for 10 minutes at room temperature, and serum samples were aliquoted and stored at –80°C until analysis. Demographic and clinical data were collected at the time of enrollment.

### Antibody titers.

Serum anti–SARS-CoV-2 spike receptor binding domain–specific (RBD-specific) antibodies were assessed in a blinded fashion at the Clinical Virology Laboratory of Hadassah Medical Center using Architect SARS-CoV-2 IgG II Quant assay (Abbott Diagnostics).

### Statistics.

Statistical analyses were performed on IBM SPSS 27 for Windows (IBM Corp.) and Prism 5.01 (GraphPad Software). Comparisons between groups (antibody concentrations and clinical continuous parameters) were analyzed with Kruskal-Wallis 1-way ANOVA, followed by Dunn’s pairwise comparisons test; alternatively, comparisons were analyzed by Mann-Whitney *U* test for nonpaired comparisons and Wilcoxon rank sum test for paired dyads (if only 2 groups were compared). Maternal–cord blood dyad antibody correlations were analyzed by Spearman’s correlation tests. Proportional data were analyzed with the Pearson χ^2^ test. Bonferroni correction for multiple comparisons was applied. All statistical tests were 2 tailed and considered significant at *P* < 0.05.

### Study approval.

Hadassah Medical Center IRB approved the study (HMO-0389-20, HMO-0274-21). Only following informed consent, participants were assigned to 1 of 2 study groups.

## Author contributions

OB, ACP, LTM, and SY designed the research studies. ACP, LTM, HAK, RF, M Kabessa, EOD, and SL conducted the experiments and collected the samples. ML, DG Wolf, ACP, and LTM acquired the data. OB, ACP, LTM, SY, and ML analyzed the data. OB, AW, M Kovo, DG Wohl, MN, SMC, ACP, LTM, and SY participated in writing the manuscript. Both ACP and LTM made a substantial contribution to the study and therefore both are listed as first co-authors. Between the two, co-first authorship was determined as follows: ACP conceived and implemented the study and was therefore listed first; LTM implemented the study and was listed second. OB was selected to last position because he also conceived the study.

## Supplementary Material

Supplemental data

ICMJE disclosure forms

## Figures and Tables

**Table 1:**
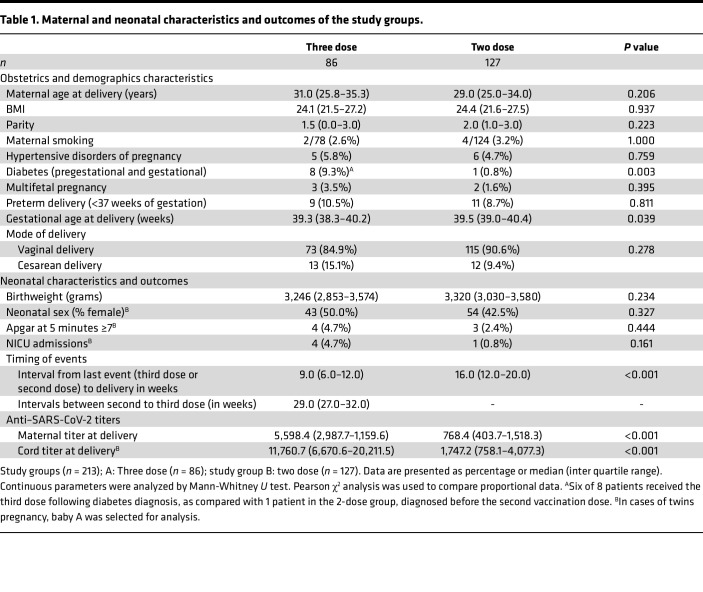
Maternal and neonatal characteristics and outcomes of the study groups.

## Version 1 01/10/2023

Electronic publication
