# Supplementary material for: Boosting maternal and neonatal anti–SARS-CoV-2 humoral immunity using a third mRNA vaccine dose
Source: JCI Insight. 2023 Jan 10;8(1):e158646. doi: 10.1172/jci.insight.158646 (PMC9870074; doi:10.1172/jci.insight.158646)
Supplement: Supplemental data [file jciinsight-8-158646-s251.pdf]

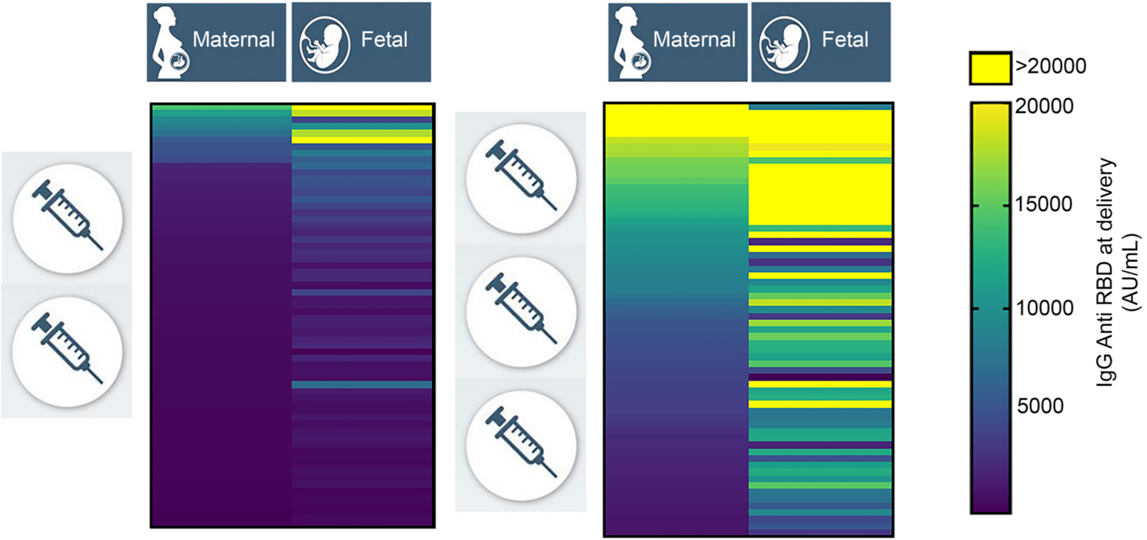

Supplemental Figure 1. Serological heat map.
The heat map was generated from the acquired anti-SARS-CoV-2 antibody titers of 64 representative maternal-cord blood dyads from each of the study groups: second dose recipients, left; third dose, right. Within each heat map, each row represents a maternal-cord blood dyad, ranked by the IgG anti-RBD level (Low, blue; high, yellow).

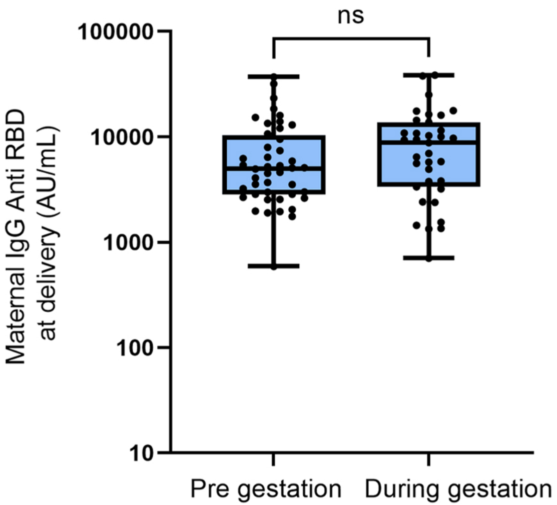

Supplemental Figure 2. Timing of initial vaccination (before vs. during gestation) and maternal humoral response to third booster dose. SARS-CoV-2 anti-RBD- specific IgG antibody titers at delivery of participants from the third vaccine group stratified by timing of the second vaccine dose (pre-gestation – 44 parturients, during gestation – 35 parturients). Significant differences for comparison were determined by Mann Whitney U test. Box and whiskers: Midline: median; Box: interquartile range; Whiskers: minimum and maximum results.

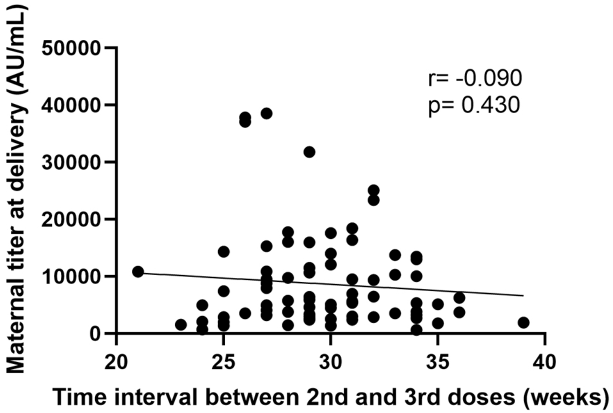

Supplemental Figure 3. Absolute time interval between two and three doses (irrespective of pregnancy) and its association with maternal titers (n = 79). Significant differences for comparison were determined by Mann Whitney U test.

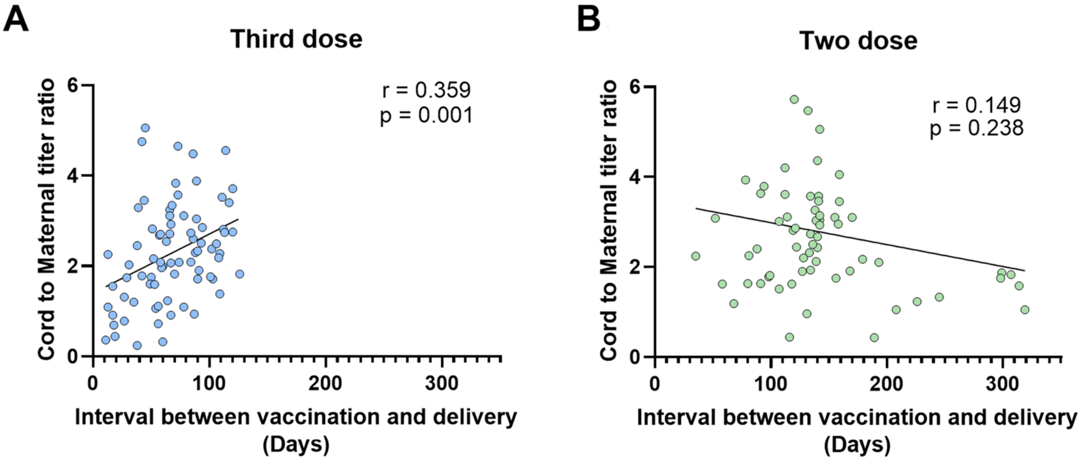

Supplemental Figure 4. Cord to maternal titer ratio (CMTR) stratified by the time between vaccination and delivery for each of the 78 dyads among the third dose patients (A) and for each of the 64 dyads among the second dose recipients (B). Significant differences for comparison were determined by Mann Whitney U test.
